# Effect of lithium chloride on the luteal steroidogenesis in gonadotropin-stimulated rat

**Published:** 2012-05

**Authors:** Maryam Khodadadi, Shiva Basavaiah, Saeid Abediankenari

**Affiliations:** 1 *Department of Studies in Zoology, University of Mysore, Manasagangotri, Mysore-6, India.*; 2 *Department of Microbiology and Immunology, Faculty of Medicine, MazandaranUniversity of Medical Sciences, Sari, Iran.*

**Keywords:** *Luteal steroidogenesis*, *Lithium chloride*, *Corpus luteum*, *Progesterone*, *Estradiol*

## Abstract

**Background:** Main function of corpus luteum is progesterone synthesis that is significantly accompanied with an increase in levels of mRNA encoding of steroidogenic enzymes known as luteal markers.

**Objective:** This study was designed to evaluate effects of lithium chloride on the release of steroid hormones and steroidogenic enzymes in gonadotropin-stimulated rats.

**Materials and Methods:** Immature 23 days old Wistar rats were divided into 10 groups; each group comprised of 8 rats, and induced with single injection of pregnant mare’s serum gonadotrophin (PMSG) and followed by single injection of human chorionic gonadotropin (hCG). Then, rats were given lithium chloride (LiCl) or saline at 12 hours post-hCG injection. Ovaries were collected in 4-hour interval from 8-24 hour post-hCG injection. Expression pattern of steroidogenic acute regulatory protein (StAR), side-chain cleavage cytochrome P450 (P450scc) and 3β-hydroxysteroid dehydrogenase (3β-HSD) genes were determined by semi-quantitative RT-PCR. In addition, serum levels of progesterone and 17β-estradiol were measured by ELISA.

**Results:** Our results showed that hCG stimulation of progesterone was markedly diminished and transcript levels of key steroidogenic enzymes were altered in the hormone-stimulated rats following LiCl treatment.

**Conclusion:** These results suggest that critical steps in the function of corpus luteum are disrupted by lithium. It is concluded that LiCl is an effective factor for suppressing of steroid genes expression.

## Introduction

“Corpus luteum (CL) is a steroidogenic gland that plays a central role in the maintenance of pregnancy. This function is carried out largely by progesterone which is the main steroid synthesized by this transient endocrine gland” (1). 

The CL produces high levels of key proteins involved in the processing of cholesterol to progesterone. The rate-limiting step in steroid hormone production, the translocation of cholesterol from the outer to the inner mitochondrial membrane, is mediated by steroidogenic acute regulatory protein (StAR) (2). 

Once cholesterol is present in the inner mitochondrial membrane, transformation of cholesterol into steroid hormones begins. This step involves the several proteins, including side-chain cleavage cytochrome P450 (P450scc) and one of the six isoforms of 3β-hydroxysteroid dehydrogenase (3β-HSD), 3βHSD-II, which is located in the smooth endoplasmic reticulum. These proteins are the sites of regulation by tropic hormones such as prolactin (PRL), LH and estradiol that are reviewed by Stocco *et al* (3). For over fifty years, a number of chemical salts of lithium have been used as a primary drug for the treatment mental disorders like mania and depression and bipolar manic-depressive psychosis (4). Despite its clinical importance, evidence suggests that lithium has side effects on reproductive and endocrine systems. Lithium chloride (LiCl) administration decreases several parameters including ovarian steroidogenic enzymes and folliculogenesis in the adult female rats (5). 

In this present study, we examined the effect of LiCl on the function of CL and the expression of steroidogenic genes in the ovary of gonadotropin-stimulated rat model.

## Materials and methods

**Animals used**

In experimental study, immature (23d-old) female albino rats of the Wistar strain were used. The animals were housed in Plexiglas cages, and kept under controlled temperature (22±2^o^C) and 12/12-hour light-dark cycle and allowed free excess to rat chow and water. The procedures were performed in accordance with institutional guidelines for animal care and use. The Research and Ethics Committee of the Department of studies in Zoology, University of Mysore, Manasagangotri approved the experimental protocol.

**Experimental design**

Female rats were divided into 10 groups. Each group comprised of 8 rats. Rats were given single intraperitoneally (i.p.) injection of 15 IU pregnant mare’s serum gonadotrophin (PMSG) (Intervet Inc., Germany) to induce follicular maturation followed by single i.p. injection of 15 IU human chorionic gonadotropin (hCG) (Intervet Inc., Germany) to induce ovulation 48h later and single i.p. injection of lithium chloride (LiCl) (250 mg/kg) (Sigma, Germany) or saline at 12h post-HCG injection (around of ovulation time). 

Rats injected only with PMSG, hCG and saline were considered as control groups. Rats were killed by spinal dislocation in 4 hours interval from 8-24 h after hCG injection. Blood was collected by cardiac puncture. Serum was separated by centrifugation and stored at -20^o^C until used for subsequent determination of hormonal parameters. The ovaries were rapidly removed, washed in a cold saline solution, snap-frozen in liquid nitrogen and stored at -80^o^C until they used in RNA extraction. 

**Hormonal assay**

Serum levels of progesterone and 17β-estradiol (E2) were determined by an ELISA kit (IBL-Hamburg, Germany) according to the manufacturer’s protocol. All samples were analyzed in duplicate.

**RNA isolation and semi-quantitative RT-PCR**

Total RNA was isolated from whole ovaries using the RNeasy mini kit (Qiagen Sciences, Germany) and first-strand complementary DNA (cDNA) was synthesized from 2 µg of total RNA using cDNA synthesis kit (Qiagen, Germany), according to the manufacturer’s protocol. Polymerase chain reaction (PCR) was performed with 2 µl of cDNA in a final volume of 25µl with rat-specific primers as previously described (5). The PCR conditions used for StAR, P450scc (CYP11A1) and 3βHSD-II were as follows: 95^o^C for 2 min, x-cycles (95^o^C for 30 sec, y^o^C for 30 sec, and 72^o^C for 60 sec), and then 72^o^C for 10 min. The extension cycle and annealing temperature are respectively shown as x and y and given in table I. Also Glyceraldehyde 3-phosphate dehydrogenase (GAPDH) was used as internal control. The PCR condition used for GAPDH was 94^o^C for 3 min, and 26 cycles of 94^o^C for 30 sec, 58^o^C for 30 sec and 72^o^C for 45 sec. PCR products were subjected to 1% agarose gel electrophoresis, stained with ethidium bromide for visualization, and quantified using Image J software (version 1.43). 

**Statistical analysis**

Experiments were repeated at least two times with eight animals per time point. Statistical analysis was performed using One-way ANOVA. Differences between means were considered significant at the p<0.05 level. These analyses were performed using SPSS software (Software program, version 13, SPSS, Inc., Chicago, USA).

## Results

**Effect of LiCl treatment on serum concentration of steroid hormones in gonadotropin-stimulated rats**

**Figure 1:**
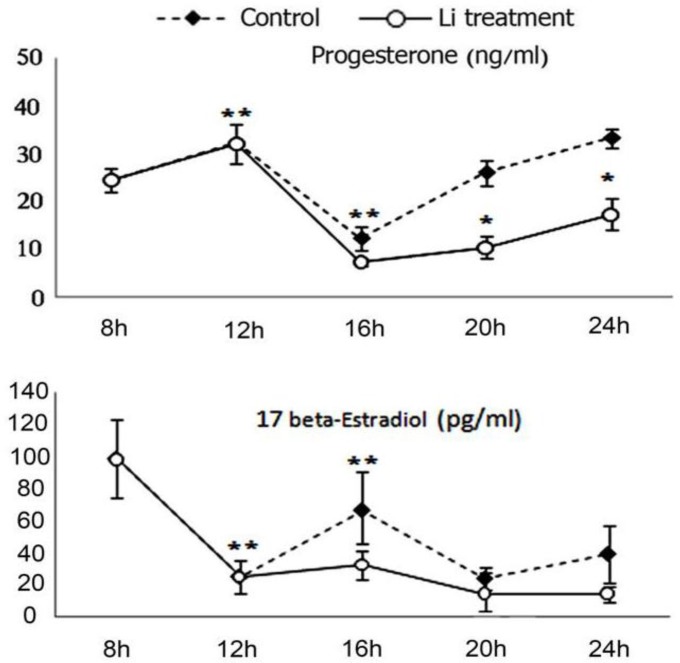
Effects of lithium chloride on the serum concentrations of progesterone and 17β-estradiol. Progesterone and 17β-estradiol serum concentrations were determined by ELISA in 4 h interval from 8 to 24 h after hCG injection. The results represent the means ± SEM of groups of eight rats *, The means ± SEM are significantly different from respective control group, p< 0.05. **, The means ± SEM are significantly different from control group obtained at 12 hours after hCG injection, p< 0.05

We evaluated the early effect of LiCl treatment on the pattern of circulating steroid hormones after ovulation in hormone-stimulated rats. In this model, ovulation approximately occurred at 12-14h post-HCG injection (The oocytes were observed by applying gentle pressure to both ends of the ampulla, placed on a slide under a stereomicroscope). Following hCG injection, serum levels of progesterone increased and the preovulatory peak of progesterone (12 h post-hCG injection) was followed by a decrease, at 16 h, and then increased between 16 and 24 h in control rats (Figure 1). Thus, a positive correlation was revealed between the serum progesterone concentrations and the *initiation of luteinization* with hCG in control rats. 

Following LiCl treatment, significant decreases were observed in progesterone concentrations, with a 2- to 3-fold decrease in 16, 20 and 24 h post-hCG injection (Figure 1). Furthermore, the peak of E_2_ was observed at 8 h that was followed by alternate decrease and increase in control rats (Figure 1). It was low, but not significant, in LiCl-treated animals *in comparison* with *control* (Figure 1).

**RT-PCR analysis of the relative expression of steroidogenic enzyme genes after LiCl treatment in gonadotropin-stimulated rats**

**Figure 2:**
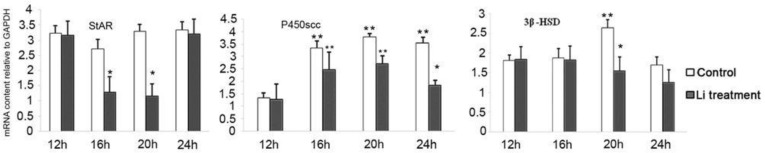
Semi-quantitative RT-PCR was performed to determine the relative expression of StAR, P450scc and 3β-HSD-ΙΙ mRNA in rat ovary during time post-hCG injection. Relative levels of mRNA were expressed as the ratio of signal intensity for the target genes relative to that for the housekeeping gene (GAPDH). The results represent the means ± SEM of groups of eight rats. *, The means ± SEM are significantly different from respective control group, p< 0.05. **, The means ± SEM are significantly different from control group obtained at 12 hours after hCG injection, p< 0.05

**Figure 3:**
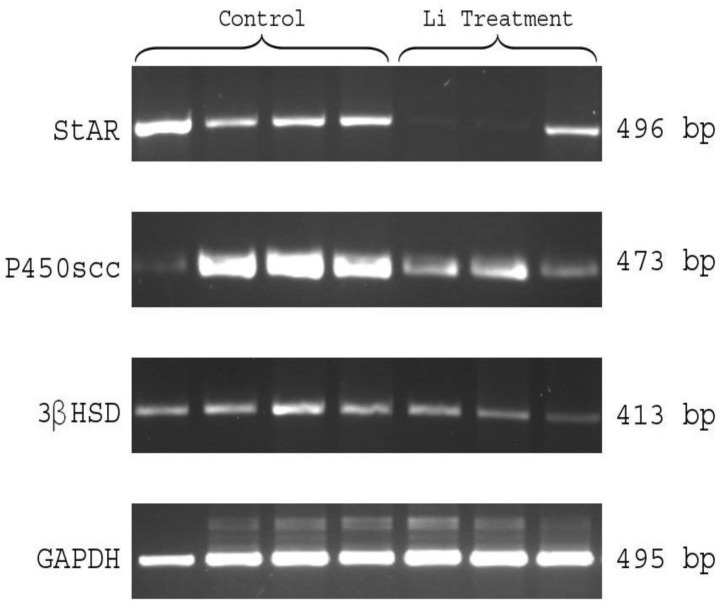
Semiquantitative RT-PCR results of StAR, P450scc and 3βHSD expression in the ovary of control and LiCl treatment rats in 4 h interval from 12 to 24 h after hCG injection

Ovaries were isolated from gonadotropin-stimulated rats in the presence or absence of LiCl in 4 h interval from 12-24 h after hCG injection and the expression of steroidogenic enzymes was determined by semi-quantitative RT-PCR. As shown in figure 2 and 3, StAR was expressed at12 h post-hCG injection, and was totally high after ovulation (16, 20 and 24 h) in control groups. Whereas, significant decreases were observed in levels of StAR mRNA at early time of luteinization following LiCl treatment, with a 2- to 2.5-fold decrease in 16 and 20 h post-HCG injection (Figure 2, 3). 

This effect was temporary and levels of StAR mRNA were equivalent to control group at 24 h post-hCG injection (Figure 2). P450scc was also expressed highly during time post-HCG injection in control groups. LiCl-treated rats had a similar pattern but, the significant decrease in P450scc mRNA levels was observed at 24 h, with a 1.9-fold decrease, in LiCl treatment group as compared with control (Figures 2, 3). On the other hand, the peak of 3βHSD-II mRNA was observed at 8h after ovulation (20 h post-HCG injection) in control rats. However, following LiCl treatment, its expression gradually decreased and the significant decrease in 3βHSD-II mRNA levels was observed at 20 h, with a 1.7-fold decrease, in LiCl treatment group as compared with control (Figures 2, 3).

**Table I:** Primer sequences and reaction conditions used in the PCR amplification of the various cDNAs

**Target mRNA**	**Forward primer(5’-3’)**	**Reverse primer(5'-3')**	**Size of roduct (bp)**	**Annealing temp.(c)**	**Extension cycles**
StAR	CATCCAGCAAGGAGAGGAAG	CGTGAGTTTGGTCTTTGAGG	496	64	25
3β-HSD	TGAAAAATGGTGGCACACTGC	TATAGTTGTAAAATGGACGCAGC	413	59	25
CYP11A1	TCAAAGCCAGCATCAAGGAG	GCAGCCTGCAATTCATACAG	473	60	26
GAPDH	CAAGGTCATCCATGACAACTTTG	GTCCACCACCCTGTTGCTGTAG	495	58	26

## Discussion

In rodents transformation of granulosa cells into luteal cells occurs within a few hours (6). After luteinization, androstenedione and estradiol is being synthesized, but luteal cells become a major site of progesterone biosynthesis (1, 3). In the present study, we observed a decrease in serum levels of E_2_ and an increase in serum levels of progesterone in response to the ovulatory dosage of hCG between 8 and 12h (Figure 1) that has been observed during rat estrous cycle (7) and indicate the follicular development and ovulation. 

Thus an inverse relationship was observed between progesterone and E_2_ before ovulation. The progesterone concentrations dramatically increased between 16 and 24 h that suggesting luteinization and progesterone biosynthesis. Although, the effect of gonadotropin treatment on the progesterone levels was attenuated by LiCl and serum progesterone levels decreased at 20 and 24 h in LiCl-treated rat in comparison with control (Figure 1). 

It is demonstrated that PRL, the pituitary hormone, affects the formation and function of CL in rat (8) and removal of the endogenous PRL results in failure of luteal development and reduced progesterone secretion by the CL in rat (9). Previous studies showed the plasma levels of gonadotropins and PRL were significantly reduced by lithium in rats and humans (10, 11). Although we have not measured PRL in our study, it is conceivable that PRL levels could be affected by lithium and led to the changes in the process of luteinization. Also, in rodents, estradiol is produced locally by luteal cells as a potent tropic hormone and progesterone secretion is directly stimulated by estradiol (12). Furthermore, the ability of the rat CL to respond to estrogen requires PRL, which stimulates the expression of the steroid receptors (ERs) (13). 

In the present study, there was no significant difference in the E_2_ serum concentration between control and LiCl-treated groups, but this data suggests that the negative feedback exerted by lithium might block ER expression during luteinization in LiCl-treated rat. Thus, the difference observed in estradiol concentration between control and LiCl- treated rats might be physiologically significant. 

On the other hand, low levels of progesterone in LiCl-treated rat could be due to change in the levels of mRNA encoding components of the luteal steroidogenic pathway, i.e., StAR, P450scc and 3β-HSD. The Formation of the CL is accompanied by a dramatic increase in the expression of these genes that is reviewed by Niswender *et al*, 2000 (14). 

The present study showed that StAR expression increased between 16 and 24 h in control rats and this increase was positively correlated with the progesterone concentration that is demonstrated in the previous study (15), while LiCl treatment was associated with a significant decrease in StAR expression at 16 and 20 h post-hCG treatment. Previous studies showed that LH stimulates StAR expression via increasing intracellular cyclic-AMP levels (cAMP) (16), while lithium inhibits cAMP formation (17). 

The effect of lithium to lower cAMP formation suggests that the decrease in StAR expression might be the consequence of changes in LH/cAMP. However, this effect was temporary and StAR mRNA expression was comparable to that of control at 24 h (Figure 2). We also observed significant decrease (p<0.05) in the expression levels of P450scc and 3βHSD-II in LiCl-treated rats after the beginning of luteinization (between 16 and 24h). In the previous studies, PRL has been shown to up-regulate the expression of P450scc and 3βHSD (18, 19). 

Also, LH increase mRNA of StAR, 3β-HSD and cytochrome P450scc expression and the concentrations of their protein in luteal cells (20, 21). It has been reported that lithium affects serum levels of LH and PRL (10, 11). Thus, the changes observed in the expression of StAR, P450scc and 3βHSD- II could be due to the effect of lithium in pituitary productions. Hypophysectomized animals could elucidate whether the effects observed of LiCl are attributable to pituitary prolactin. Additionally the reduction observed in progesterone concentrations at 20 and 24 h post-hCG injection by LiCl treatment could be due to the low expression of these steroidogenic genes in LiCl-treated rat.

It is concluded that this study has provided evidences that during the period of post-hCG treatment, lithium is able to decrease serum levels of progesterone and alter the expression of StAR, P450scc and 3β-HSD-II genes that are involved in processes of steroidogenesis. Since, StAR, P450scc and 3β-HSD-II genes are almost exclusively expressed in the CL, the decrease in expression of these genes could be responsible for the decrease in levels of circulating progesterone following LiCl treatment. The action of lithium could involve pituitary hormones, viz. PRL and LH which regulate CL formation and differentiation. Our results suggest that further investigations are required to knowledge of the role of PRL and E_2_ in luteinization. 
